# Supplementary material for: A Likelihood Approach for Real-Time Calibration of Stochastic Compartmental Epidemic Models
Source: PLoS Comput Biol. 2017 Jan 17;13(1):e1005257. doi: 10.1371/journal.pcbi.1005257 (PMC5240920; doi:10.1371/journal.pcbi.1005257)
Supplement: S1 File — (TAR.GZ) [file pcbi.1005257.s014.tar.gz › HSPH_Online-SI-Revision/PF08/n100-severe/n100-severe_PF_table-8.pdf]

1	R0	{1.65278, 9.06793}	{1.7885, 18.9067}	{{1.57723, 14.6163}, {1.92404, 20.}}	{9.80297, 108.501}	inf	990.	1581.49	{1264.5, 1835.12}	59.7463	0.198266	newly	963.	677.2	{533., 806.}	29.9273	0.159689	Reff	1.60825	1.73906	{1.53615, 1.8698}	9.67662	ar	66 342.	76 804.1	{67 105., 82 099.}	16.1788
2	R0	{1.4321, 3.80644}	{2.01364, 16.6977}	{{1.49153, 11.3229}, {2.64126, 20.}}	{40.6956, 338.67}	inf	86.	365.778	{190.281, 595.718}	325.323	0.582054	newly	223.	195.56	{102., 333.}	39.13	0.198001	Reff	1.42869	1.99087	{1.48285, 2.61758}	39.5414	ar	54 217.	78 206.5	{60 271., 93 200.}	44.3193
3	R0	{1.68388, 4.76028}	{2.01039, 16.4632}	{{1.37352, 8.46592}, {2.96604, 20.}}	{30.8623, 245.845}	inf	40.	243.533	{93.9624, 419.817}	508.832	0.708909	newly	115.	134.35	{48., 229.}	54.913	0.227881	Reff	1.68211	1.98485	{1.35027, 2.95067}	30.0721	ar	68 299.	73 712.8	{49 701., 94 630.}	24.0511
4	R0	{1.66014, 6.95383}	{1.95707, 16.4903}	{{1.48182, 9.36348}, {2.63296, 20.}}	{24.5482, 137.14}	inf	267.	496.161	{270.41, 844.427}	87.9669	0.237241	newly	379.	260.68	{139., 394.}	35.2507	0.213378	Reff	1.65028	1.93151	{1.45828, 2.5969}	24.5014	ar	66 820.	75 592.9	{58 402., 93 524.}	19.4608
5	R0	{1.69984, 11.2038}	{1.91064, 18.086}	{{1.61008, 14.2707}, {2.16336, 20.}}	{14.4559, 61.4274}	inf	1644.	2459.51	{1668.65, 3155.84}	50.5015	0.168521	newly	1210.	1099.76	{894., 1335.}	13.2463	0.0628812	Reff	1.6312	1.7987	{1.54852, 2.00103}	12.062	ar	68 462.	80 680.4	{69 890., 88 307.}	18.1378
6	R0	{1.65876, 6.66217}	{1.93318, 17.543}	{{1.50299, 12.7166}, {2.65317, 20.}}	{22.6623, 163.322}	inf	148.	415.878	{199.879, 790.44}	180.998	0.398637	newly	251.	207.43	{84., 383.}	41.8127	0.226069	Reff	1.65251	1.91283	{1.48907, 2.61208}	22.1523	ar	67 105.	75 213.4	{60 579., 93 254.}	18.2338
7	R0	{1.69966, 5.89996}	{1.9615, 16.6472}	{{1.48501, 9.33058}, {2.65263, 20.}}	{24.1547, 182.158}	inf	95.	328.557	{200.069, 509.352}	245.85	0.505627	newly	161.	178.45	{86., 318.}	40.4161	0.169989	Reff	1.69633	1.9453	{1.46632, 2.62856}	23.827	ar	69 396.	75 790.7	{59 610., 93 432.}	18.3771
8	R0	{1.53428, 6.3734}	{1.87526, 17.6371}	{{1.52253, 14.1605}, {2.11575, 20.}}	{23.0959, 176.73}	inf	389.	953.89	{647.083, 1226.83}	145.216	0.376112	newly	553.	451.27	{304., 600.}	23.5859	0.122498	Reff	1.51552	1.83135	{1.51008, 2.06843}	21.6184	ar	59 855.	77 648.	{63 320., 85 643.}	30.2729
9	R0	{1.58939, 3.48608}	{1.96104, 16.1366}	{{1.30712, 8.21437}, {2.75521, 20.}}	{32.3311, 362.886}	inf	10.	239.535	{94.4452, 422.727}	2295.35	1.31071	newly	29.	131.83	{52., 222.}	356.241	0.59489	Reff	1.58888	1.93908	{1.29205, 2.74461}	31.3834	ar	62 575.	72 570.4	{44 778., 93 013.}	29.5199
10	R0	{1.58528, 5.5593}	{1.82738, 20.}	{{1.7798, 20.}, {1.88274, 20.}}	{15.2721, 259.758}	inf	179.	906.087	{770.099, 1044.66}	406.194	0.701502	newly	327.	361.77	{305., 424.}	13.581	0.0541724	Reff	1.57528	1.80792	{1.76035, 1.85848}	14.7687	ar	63 039.	78 890.5	{76 721., 80 963.}	25.1456
11	R0	{1.58305, 4.62721}	{1.8554, 15.8817}	{{1.30323, 8.45965}, {2.73265, 20.}}	{28.1345, 243.224}	inf	50.	308.725	{131.911, 525.707}	517.451	0.722806	newly	133.	170.5	{69., 312.}	56.2857	0.20475	Reff	1.57961	1.82549	{1.29916, 2.6708}	27.4222	ar	63 222.	68 328.3	{44 088., 92 227.}	26.5015
12	R0	{1.70234, 9.34626}	{1.8358, 19.9467}	{{1.70386, 20.}, {1.89638, 20.}}	{8.08382, 113.419}	inf	872.	1402.07	{1206.2, 1622.93}	60.7881	0.203636	newly	836.	564.76	{471., 651.}	32.445	0.173581	Reff	1.66487	1.79636	{1.66611, 1.8584}	8.12554	ar	68 213.	79 077.8	{74 534., 81 748.}	15.9278
13	R0	{1.68556, 9.74954}	{1.88375, 18.9128}	{{1.62211, 15.5118}, {2.10522, 20.}}	{13.4002, 93.9863}	inf	1232.	2083.3	{1613.22, 2798.36}	69.4475	0.21908	newly	1100.	893.43	{691., 1150.}	20.8955	0.107491	Reff	1.63051	1.80108	{1.57853, 1.94908}	11.9639	ar	68 426.	79 880.	{70 869., 86 442.}	17.435
14	R0	{1.59876, 9.78897}	{1.80604, 19.3057}	{{1.63152, 16.1426}, {1.92776, 20.}}	{13.3432, 97.2191}	inf	1006.	1671.84	{1393.47, 2010.04}	66.1867	0.215734	newly	885.	702.25	{557., 857.}	21.452	0.109606	Reff	1.55367	1.7569	{1.60225, 1.88373}	13.4288	ar	63 553.	77 894.9	{70 972., 82 796.}	22.5848
15	R0	{1.43577, 5.12819}	{1.79844, 19.9092}	{{1.66592, 20.}, {1.87658, 20.}}	{25.2596, 288.231}	inf	217.	769.195	{629.581, 864.089}	254.468	0.545637	newly	384.	308.53	{247., 356.}	21.1536	0.106186	Reff	1.42469	1.78463	{1.65481, 1.86098}	25.2646	ar	53 259.	77 710.5	{72 252., 81 044.}	45.9105
16	R0	{1.58866, 8.73191}	{1.83082, 19.8889}	{{1.69969, 20.}, {1.89611, 20.}}	{15.253, 127.772}	inf	604.	1075.52	{893.952, 1297.65}	78.0662	0.245026	newly	587.	436.56	{354., 539.}	27.0426	0.141228	Reff	1.56477	1.80182	{1.67698, 1.8658}	15.155	ar	64 349.	79 016.1	{74 230., 81 830.}	22.7931
17	R0	{1.43012, 3.8163}	{2.12204, 17.5058}	{{1.51048, 13.0237}, {2.88592, 20.}}	{48.6283, 358.711}	inf	64.	413.906	{191.78, 767.982}	546.729	0.752556	newly	190.	211.16	{97., 405.}	44.1579	0.179347	Reff	1.42695	2.08799	{1.50171, 2.66553}	46.567	ar	53 353.	80 167.5	{60 930., 94 726.}	50.5644
18	R0	{1.47965, 5.16472}	{1.87382, 16.9535}	{{1.52785, 12.8459}, {2.60233, 20.}}	{27.1059, 228.257}	inf	233.	552.563	{298.909, 997.549}	137.954	0.339077	newly	443.	277.69	{145., 476.}	42.1919	0.265937	Reff	1.47015	1.84379	{1.48555, 2.55563}	25.9964	ar	56 086.	74 284.3	{61 187., 92 962.}	32.9086
19	R0	{1.57191, 5.2314}	{1.87213, 17.502}	{{1.52003, 13.4871}, {2.2767, 20.}}	{20.6508, 234.556}	inf	225.	507.164	{310.051, 793.541}	126.069	0.32313	newly	437.	245.27	{135., 394.}	47.0412	0.300848	Reff	1.563	1.83708	{1.49443, 2.24209}	19.7095	ar	63 119.	75 165.7	{61 647., 87 942.}	20.694
20	R0	{1.54435, 5.32911}	{1.8248, 19.8281}	{{1.69577, 19.6845}, {1.93096, 20.}}	{18.2041, 272.072}	inf	191.	628.212	{473.871, 910.615}	228.907	0.503939	newly	372.	256.87	{190., 388.}	35.4973	0.195114	Reff	1.53601	1.81182	{1.68961, 1.92279}	17.9976	ar	61 870.	78 448.6	{73 978., 82 340.}	26.7959
21	R0	{1.57932, 4.90031}	{2.00475, 17.396}	{{1.51697, 10.9442}, {2.65714, 20.}}	{29.0579, 254.998}	inf	57.	320.873	{169.892, 547.393}	462.934	0.707086	newly	102.	163.8	{80., 270.}	71.4902	0.215424	Reff	1.57657	1.98683	{1.50109, 2.63034}	28.422	ar	63 595.	77 625.9	{61 827., 93 370.}	24.229
22	R0	{1.47044, 4.07694}	{1.834, 17.7632}	{{1.5272, 14.0974}, {2.05592, 20.}}	{25.2982, 335.699}	inf	83.	461.706	{261.33, 612.757}	456.272	0.714816	newly	207.	218.58	{112., 299.}	31.8841	0.136444	Reff	1.46579	1.80553	{1.51356, 2.02108}	23.7625	ar	55 895.	74 078.6	{61 437., 82 618.}	33.2964
23	R0	{1.64997, 3.34546}	{1.99042, 14.9957}	{{1.37515, 7.88336}, {2.96976, 20.}}	{30.5218, 348.241}	inf	9.	215.591	{81.9509, 386.134}	2295.46	1.30768	newly	51.	153.91	{48., 270.}	206.647	0.376805	Reff	1.64943	1.95874	{1.34844, 2.94359}	29.4263	ar	66 404.	72 948.2	{49 700., 94 866.}	24.5223
24	R0	{1.67405, 4.50254}	{1.92059, 13.8861}	{{1.18976, 6.16453}, {2.75598, 19.5242}}	{31.5662, 209.505}	inf	14.	171.233	{16.5687, 388.95}	1137.57	0.980319	newly	40.	118.7	{6., 271.}	226.35	0.594599	Reff	1.6737	1.8705	{1.15086, 2.7524}	30.6609	ar	68 017.	63 558.8	{17 527., 91 955.}	34.1853
25	R0	{1.50663, 3.1286}	{1.92589, 13.8085}	{{1.19018, 6.16453}, {2.75598, 19.5242}}	{37.8824, 341.363}	inf	3.	164.358	{8.26673, 388.95}	5387.43	1.52332	newly	20.	116.21	{6., 283.}	505.25	0.777781	Reff	1.50652	1.8767	{1.15086, 2.7524}	36.3058	ar	58 823.	64 145.3	{28 247., 91 955.}	42.1939
26	R0	{1.69822, 6.34992}	{1.81204, 19.5496}	{{1.62029, 18.792}, {2.06285, 20.}}	{8.37371, 207.871}	inf	141.	461.249	{341.092, 696.076}	227.127	0.496947	newly	238.	192.03	{127., 292.}	30.9874	0.159397	Reff	1.69328	1.7899	{1.61373, 1.97061}	7.43327	ar	69 816.	76 384.5	{68 591., 84 241.}	10.5963
27	R0	{1.55869, 4.92988}	{1.85341, 18.7746}	{{1.58754, 15.3674}, {2.11503, 20.}}	{19.3499, 280.833}	inf	86.	426.642	{270.646, 589.098}	396.095	0.674191	newly	214.	190.88	{103., 273.}	28.6729	0.14466	Reff	1.55537	1.83646	{1.58271, 2.08311}	18.5175	ar	62 344.	77 150.7	{66 435., 85 577.}	23.9428
28	R0	{1.46085, 6.06899}	{2.12399, 16.7958}	{{1.52828, 9.505}, {2.67728, 20.}}	{45.7464, 176.749}	inf	547.	1276.58	{643.384, 1921.45}	134.212	0.32871	newly	795.	657.94	{334., 891.}	24.317	0.136754	Reff	1.43891	2.06777	{1.48205, 2.60774}	44.1364	ar	55 484.	80 310.9	{61 606., 93 483.}	44.9279
29	R0	{1.63017, 6.25164}	{1.84278, 17.5143}	{{1.52171, 13.93}, {2.0431, 20.}}	{16.3383, 180.156}	inf	248.	462.683	{252.825, 608.077}	89.6122	0.259131	newly	360.	224.63	{111., 317.}	40.8417	0.253409	Reff	1.6206	1.81554	{1.49917, 2.02285}	15.6496	ar	66 231.	74 613.3	{62 044., 82 791.}	16.2008
30	R0	{1.45173, 5.68165}	{1.80079, 19.999}	{{1.72574, 20.}, {1.86612, 20.}}	{24.0444, 251.993}	inf	352.	937.524	{800.183, 1112.52}	166.342	0.42222	newly	541.	375.59	{327., 452.}	30.5749	0.162159	Reff	1.43681	1.77852	{1.7059, 1.84228}	23.7828	ar	55 280.	77 652.4	{74 786., 80 090.}	40.4711
31	R0	{1.55808, 2.77316}	{1.93258, 13.8204}	{{1.19018, 6.16453}, {2.85956, 19.5242}}	{35.9262, 398.363}	inf	4.	164.751	{8.26673, 388.95}	4028.4	1.41374	newly	23.	116.41	{6., 283.}	431.87	0.739283	Reff	1.55797	1.8821	{1.15086, 2.7524}	34.5428	ar	60 940.	64 221.5	{28 247., 92 658.}	39.8674
32	R0	{1.56797, 3.90327}	{2.02242, 16.648}	{{1.3704, 10.9997}, {2.96479, 20.}}	{34.5639, 326.515}	inf	24.	234.611	{85.1425, 417.827}	877.546	0.920311	newly	62.	130.66	{46., 232.}	123.032	0.307375	Reff	1.56717	1.99333	{1.36576, 2.95018}	33.4604	ar	62 228.	74 576.4	{49 813., 94 672.}	28.9556
33	R0	{1.70027, 5.43261}	{1.98487, 15.8499}	{{1.32142, 8.13727}, {3., 20.}}	{30.7288, 191.755}	inf	66.	243.228	{93.2483, 408.749}	269.269	0.502058	newly	163.	140.51	{55., 254.}	42.7301	0.237723	Reff	1.69787	1.96111	{1.31165, 2.95453}	30.3195	ar	68 798.	72 322.7	{44 968., 94 686.}	25.083
34	R0	{1.61668, 6.86192}	{1.79322, 19.9993}	{{1.74867, 20.}, {1.84498, 20.}}	{10.9199, 191.453}	inf	325.	1022.15	{883.814, 1200.61}	214.508	0.494264	newly	465.	407.89	{347., 480.}	14.6602	0.0704646	Reff	1.60218	1.76672	{1.72616, 1.81137}	10.2697	ar	64 693.	77 594.5	{75 937., 79 275.}	19.9426
35	R0	{1.55955, 3.45071}	{2.12208, 15.8331}	{{1.39153, 8.42891}, {3., 20.}}	{40.4919, 358.837}	inf	11.	229.545	{84.8109, 391.674}	1986.77	1.22655	newly	53.	155.66	{43., 373.}	201.962	0.362992	Reff	1.55925	2.09699	{1.39015, 2.96016}	39.2541	ar	61 783.	77 343.1	{52 120., 95 330.}	32.2071
36	R0	{1.65068, 4.91307}	{2.11185, 17.8229}	{{1.52822, 12.3838}, {2.6951, 20.}}	{31.2553, 262.764}	inf	26.	312.235	{168.568, 534.034}	1100.9	1.04063	newly	80.	160.27	{73., 261.}	106.138	0.277466	Reff	1.64972	2.09413	{1.49813, 2.66921}	30.7084	ar	66 453.	79 974.5	{62 287., 94 516.}	23.4619
37	R0	{1.46131, 3.06714}	{2.01994, 14.8652}	{{1.35605, 7.87158}, {2.86216, 20.}}	{41.4725, 384.658}	inf	18.	286.035	{98.8877, 509.926}	1489.09	1.11752	newly	72.	178.6	{58., 311.}	156.278	0.358673	Reff	1.45985	1.98113	{1.336, 2.77498}	39.604	ar	56 452.	74 434.8	{47 622., 93 660.}	38.7212
38	R0	{1.71034, 5.2546}	{2.04238, 17.3913}	{{1.4954, 13.0258}, {2.67715, 20.}}	{26.1585, 230.973}	inf	60.	318.948	{172.838, 508.915}	431.581	0.681285	newly	144.	165.99	{82., 245.}	44.5625	0.167566	Reff	1.70807	2.02584	{1.47619, 2.66429}	25.9481	ar	69 816.	78 249.2	{59 425., 93 712.}	19.1968
39	R0	{1.43565, 5.57989}	{1.9111, 16.8434}	{{1.60312, 13.9675}, {2.28543, 20.}}	{33.1177, 201.86}	inf	400.	938.367	{609.955, 1550.17}	134.592	0.346625	newly	680.	474.87	{308., 744.}	36.5809	0.204572	Reff	1.4178	1.87155	{1.57671, 2.23999}	32.0036	ar	54 407.	78 049.7	{68 096., 89 276.}	43.4552
40	R0	{1.40327, 4.00764}	{1.97351, 15.4406}	{{1.30991, 7.87829}, {2.92741, 20.}}	{42.9561, 285.28}	inf	122.	484.811	{184.091, 794.817}	297.413	0.53702	newly	269.	282.37	{106., 487.}	44.7026	0.198506	Reff	1.39731	1.9251	{1.29091, 2.89939}	40.7936	ar	51 976.	72 136.2	{44 679., 94 630.}	44.3363
41	R0	{1.63612, 4.26741}	{2.11646, 15.199}	{{1.37294, 7.43348}, {2.85763, 20.}}	{35.8238, 256.181}	inf	38.	257.041	{103.046, 485.035}	576.423	0.750722	newly	108.	194.81	{49., 389.}	106.472	0.27062	Reff	1.6346	2.07874	{1.36917, 2.83991}	34.3907	ar	65 029.	77 346.6	{49 922., 94 617.}	28.6621
42	R0	{1.57426, 6.50271}	{1.82717, 19.9993}	{{1.69814, 20.}, {1.90446, 20.}}	{16.0653, 207.553}	inf	287.	776.988	{620.563, 949.029}	170.727	0.426731	newly	463.	314.21	{247., 385.}	32.581	0.176938	Reff	1.56141	1.81161	{1.68519, 1.88663}	16.0239	ar	62 522.	78 600.5	{73 439., 81 622.}	25.7165
43	R0	{1.37818, 5.37636}	{1.8271, 19.6194}	{{1.67774, 19.6024}, {1.9133, 20.}}	{32.5731, 264.919}	inf	406.	1166.76	{969.053, 1361.34}	187.38	0.453389	newly	728.	481.74	{378., 607.}	34.2802	0.188903	Reff	1.35702	1.79642	{1.64953, 1.88488}	32.3791	ar	50 098.	78 602.1	{72 594., 81 994.}	56.8967
44	R0	{1.66606, 4.92641}	{1.99525, 15.8763}	{{1.36261, 8.3758}, {2.77832, 20.}}	{30.9596, 222.268}	inf	32.	249.596	{99.0617, 419.817}	679.987	0.827455	newly	79.	143.51	{54., 255.}	95.2785	0.270495	Reff	1.66501	1.97086	{1.35014, 2.76558}	30.1822	ar	67 066.	73 227.	{48 631., 93 218.}	25.3378
45	R0	{1.623, 5.9449}	{1.94845, 17.53}	{{1.51084, 13.5567}, {2.62852, 20.}}	{23.5488, 194.874}	inf	133.	420.696	{208.376, 708.289}	216.39	0.449375	newly	254.	208.34	{81., 342.}	37.7795	0.212265	Reff	1.61652	1.92576	{1.50462, 2.61168}	22.9461	ar	64 554.	76 287.1	{60 860., 92 927.}	20.8401
46	R0	{1.65235, 5.51234}	{1.8758, 19.1109}	{{1.62776, 16.1608}, {2.1104, 20.}}	{14.3865, 246.693}	inf	70.	404.787	{251.912, 547.199}	478.267	0.74379	newly	154.	176.37	{105., 245.}	35.4221	0.14038	Reff	1.64944	1.85373	{1.62437, 2.07545}	13.2642	ar	66 509.	78 173.9	{69 658., 85 247.}	17.7531
47	R0	{1.62138, 8.82208}	{2.04331, 18.8616}	{{1.69902, 15.7834}, {2.22336, 20.}}	{26.2466, 113.8}	inf	1152.	2212.29	{1663.11, 2634.13}	92.0394	0.275747	newly	1173.	981.91	{798., 1145.}	17.5797	0.0875455	Reff	1.57308	1.94716	{1.65705, 2.10694}	23.9542	ar	65 598.	85 075.9	{74 687., 89 798.}	29.6929
48	R0	{1.64073, 6.69261}	{1.98841, 17.4859}	{{1.51238, 12.7866}, {2.67851, 20.}}	{25.721, 161.272}	inf	217.	509.942	{261.229, 819.087}	136.023	0.330043	newly	314.	253.54	{123., 386.}	35.6879	0.197279	Reff	1.63171	1.96425	{1.49483, 2.65818}	25.6705	ar	66 438.	76 896.	{61 589., 93 351.}	19.484
49	R0	{1.65556, 3.796}	{1.92155, 13.8392}	{{1.19018, 6.16453}, {2.75598, 19.5242}}	{32.0893, 265.134}	inf	5.	167.209	{16.5687, 362.69}	3254.54	1.34495	newly	9.	116.88	{6., 271.}	1214.67	1.03115	Reff	1.65538	1.87201	{1.15086, 2.7524}	31.0863	ar	66 575.	63 721.2	{27 943., 91 955.}	35.0982
50	R0	{1.56828, 3.65713}	{1.99997, 15.3049}	{{1.37043, 8.4262}, {2.83103, 20.}}	{33.775, 318.496}	inf	37.	243.698	{87.713, 401.975}	558.644	0.739769	newly	103.	152.34	{49., 243.}	74.	0.237474	Reff	1.56703	1.97758	{1.36924, 2.81583}	32.8312	ar	62 761.	73 961.	{49 544., 94 519.}	28.9209
